# Factors Affecting Communication Outcomes for Deaf and Multilingual Learners: A Systematic Review

**DOI:** 10.1111/1460-6984.70191

**Published:** 2026-01-28

**Authors:** Elizabeth Kilmartin, Paul Conroy, Janine Owens

**Affiliations:** ^1^ Northern Care Alliance NHS Foundation Trust Mayo Building, Salford Royal, Stott Lane, Salford M6 8HD UK; ^2^ School of Linguistic, Speech and Communication Sciences Trinity College The University of Dublin Dublin Ireland; ^3^ School of Health Sciences, Division of Nursing, Midwifery and Social Work University of Manchester Manchester UK

**Keywords:** communication, deaf, multilingual, outcomes

## Abstract

**Background:**

Deaf and hard of hearing (DHH) children who are exposed to more than one spoken language can be described as deaf and multilingual learners (DMLs). Increased globalisation and technological advancements in hearing amplification mean an increasing number of children who are DHH access more than one spoken language (with and without the presence of signed languages). A growing body of evidence suggests that DMLs can develop competency in more than one spoken language. However, critical evaluation of factors contributing to positive outcomes appears limited.

**Aims:**

The purpose of this systematic review was to identify factors that facilitate and inhibit the development of listening, speech, and language skills in more than one spoken language for DMLs.

**Methods:**

Following PRISMA guidelines, searches of seven databases were conducted. The methodological quality of the studies used the Quality Assessment with Diverse Studies (QuADS) tool. Seventeen studies met the inclusion criteria. All studies measured outcomes in a home language and the majority community language. Forty‐two different outcome measurement/assessment tools were used, making comparison across the studies difficult.

**Main Contribution:**

A range of factors emerged from the interventions, which supported or inhibited the outcomes for DMLs. The results of this review provide insights for practitioners working with DMLs into the factors that promote spoken multilingual language learning and factors that limit positive listening, speech, and language outcomes. The results also contribute to recommendations for researchers to enable the multilingual outcome measurement for DML studies.

**Conclusions:**

The listening, speech, and language outcomes of DMLs are enhanced through interventions in each of the spoken languages to which the child is exposed. This is facilitated by increased engagement of parents who are encouraged to maintain their home language. Furthermore, culturally inclusive practices promote multilingual language development in DMLs and should be included in informing policy decision‐making.

**WHAT THIS PAPER ADDS:**

*What is already known on the subject*

- The term deaf and multilingual learners (DMLs) encompasses deaf learners who are exposed to and learning more than one spoken and/or signed language. The focus of this paper is on deaf and hard of hearing (DHH) children in environments where more than one spoken language is used. There is a growing body of literature focusing on the listening, speech, and language outcomes of DMLs. Much of the literature focuses on communication outcomes in one language (namely the majority community language). There are few studies that measure outcomes in more than one spoken language for DMLs.

*What this study adds to existing knowledge*

- This paper reviews studies that have measured the listening, speech and language outcomes of DMLs in more than one spoken language. Typically, the majority community language and a different home language (spoken). The results reveal that interventions provided in each language heard and spoken by the DML and their family acts as a facilitator for improved outcomes in each language. Interventions provided in one language only are a barrier to positive multilingual language outcomes. Language exposure in the home language was also a facilitator for positive home language outcomes. Reduced parental engagement and interventions in only one language inhibited multilingual outcomes for DMLs.

*What are the practical and clinical implications of this work?*

- The paper provides guidance for practitioners when supporting the communication development of DMLs. The provision of listening, speech, and language interventions in each of the languages spoken by the DML and their family facilitates language outcomes. Interventions that are culturally and linguistically adapted and enhance parental engagement also enhance multilingual outcomes. This paper provides evidence to support strategic planning for hearing services to meet the needs of DMLs.

## 1 Introduction

The focus of this paper is on deaf and hard of hearing (DHH) children in environments where more than one spoken language is used. It will use the definition of DHH children raised within multilingual families and communities as previous authors have used: ‘DHH children in environments where more than one spoken language is used are referred to as d/Deaf and multilingual learners (DMLs)’ (Crowe and Guiberson, 2021, 70). However, multilingualism for DHH learners can occur in numerous ways. These ways include (a) learners who use a spoken language at home that differs from the majority community spoken language; (b) a signed language at home that differs from the majority community spoken language; (c) a signed language at home that differs from the majority community signed language and majority community spoken language (Cannon et al. 2016). This paper will focus on studies of learning spoken languages in the context of spoken language multilingualism (that is, DHH learners using a spoken language at home which differs from the majority community language).

DMLs are a complex, heterogeneous and growing learning population (Baker and Scott 2016; de Diego‐Lazaro et al., 2021; Mahon et al. 2011; Pizzo and Chilvers 2016). DHH children may use a signed language as their main form of communication or use a signed language alongside spoken language (bimodal or sign multilingualism). A comparatively small body of literature focuses on the learning of speech and spoken language of DMLs (Cannon et al. 2016; Crowe and Guiberson 2021).

Heterogeneity poses a challenge for researchers because multiple variables influence DHH children's listening, speech, language, learning and education outcomes. These factors include aetiology of hearing loss, age of diagnosis, age of amplification fitting, age of entry into intervention programs, cognitive ability, comorbidity, socio‐economic status, maternal education, communication mode (Archbold 2010; Crowe et al. 2012; Dall et al. 2022; Yoshinaga‐Itano 2003). Heterogeneity for DMLs relates not only to aspects of their deafness, but also to their exposure to different languages. This can vary in terms of timing, perceived status of the languages to which they are exposed and quantity and quality of language exposure (de Houwer 2021). Within hearing children, smaller vocabularies in the dominant community language, compared to their monolingual peers are a factor in multilingualism (McLeod et al. 2016). One essential characteristic of effective (signed or spoken) language acquisition is that it helps young children interact with their social worlds, in the process developing a sense of self and their place in social environments (Hintermair 2016). However, little is known as to whether this applies to DMLs and how effective interventions are for them.

The DHH children are often born to hearing parents, who have little experience of deafness and the communication decisions accompanying raising a deaf child (National Deaf Children's Society [NDCS] 2016; Terry 2023). Hearing parents of DMLs are required to make decisions about the languages (spoken and/or signed) they will use with their deaf child (Wright et al. 2023). However, hearing parents have successfully negotiated the hearing world and the only experience of the d/Deaf world is through their child. Therefore, they know little about deafness and the factors that influence families' experience of navigating pathways for deaf children through health and education services (Terry 2023). There is a lack of support for parents, therefore they are often reliant on professional guidance (Moeller et al. 2013). Professional advice for families of DMLs previously focused on developing the majority community language, because of a common‐sense assumption, lacking in any evidence base, that developing two or more spoken languages could be too challenging or unachievable (Crowe and Guiberson 2021). The evidence, that is more recent suggests that multilingual language outcomes are achievable and necessary for DMLs growing up within a multilingual family and/or community (Benítez‐Barrera et al. 2023; Crowe et al. 2012; Crowe and McLeod 2014; McConkey Robbins et al. 2004). The family unit provides a critical role in forming the child's linguistic environment because of the exposure to the languages that they speak or sign (Schwartz and Verschik 2013). Children also learn how to interact with the people around them through social interaction models provided by family members. Therefore, excluding a DML from developing both home and majority community language(s) potentially marginalises and prevents them from forming deep, rich and meaningful relationships with any or all their family members (de Diego‐Lazaro et al. 2018; Kohnert et al. 2005). This influences their identity and sense of self. Research argues in favour of the moral and ethical responsibility of practitioners to support the social function that multilingualism serves in ensuring the cultural unity of the family and developing identity of multilingual children (including DMLs) (Pert 2023).

Providing equitable services for families from diverse ethnic, cultural and linguistic backgrounds presents a challenge for hearing, health and educational services (Mennen and Stansfield 2006). For example, professionals delivering care to children who are DHH, and their families may not share the same culture or language (Bedoin 2019). This is because the workforce is mostly part of the cultural and linguistic majority (Mennen and Stansfield 2006). An additional challenge facing professionals working with DMLs and their families appears to be the limited research on outcomes (Crowe and Guiberson 2021; Vukkadala et al. 2018).

Globalisation and migration are increasing alongside linguistic diversity, particularly for majority monolingual English speaking countries (Grandpierre et al. 2019; Cannon et al. 2016). In England, the Consortium for Research into Deaf Education (CRIDE) reported that 16% of deaf children and young people used a language other than English at home, an increase from 14% in 2021 (CRIDE 2023). However, there appears to be absence of evidence‐based interventions for use with DMLs (Crowe and Guiberson 2019). Crowe and McLeod (2014) carried out a systematic review in this area. The results identified factors which influence outcomes in languages other than English, but a comparison of factors across languages was not possible.

The aim of this study is to identify factors that affect the listening, speech and language outcomes in each language used by DMLs as represented in the current literature.

## 2 Methods

This systematic review aims to uncover international evidence on the impact of factors for DMLs, answer clinically meaningful questions and provide evidence to confirm and inform practice (Munn et al. 2018). The review adheres to the guidelines provided by the Preferred Reporting Items for Systematic Reviews and Meta‐Analyses [PRISMA] (Page et al. 2021). Specifically, the paper addresses the following question:

What factors support or inhibit the listening, speech, and language outcomes of deaf and multilingual learners?

Registration of the study CRD42023430967 used the international prospective register of systematic reviews (PROSPERO) https://www.crd.york.ac.uk/PROSPERO

## 3 Search

Two researchers searched the following databases: MEDLINE, EMBASE, PsycINFO (all via OVID), CINAHL (EBSCO), Linguistics and Language Behaviour Abstracts (LLBA) though ProQuest, and an additional Google Scholar search. Forward and backward searching of retrieved records identified through initial searches meeting eligibility criteria identified any additional relevant literature for inclusion. We excluded research before 2001, which was the start of newborn hearing screening, in the United Kingdom and because of resource limitations in languages of publication other than English. Searching occurred up to 19th July 2025.

### 3.1 Search Terms Employed

The search strategy employed the following facets, alongside Boolean operators:

Children OR adolescents

AND

Deaf OR d/deaf OR hearing loss OR hearing impair* OR hearing disorder OR hard of hearing.

AND

Multilingual* OR bilingual* OR plurilingual* OR dual language OR heritage language* OR second language learner* OR dual language learner* OR multicultural class*

It also used MeSH (Medical Subject Headings) terms to expand the search.

The researchers used the search terms ‘hearing loss’, ‘hearing impair*’, ‘hearing disorder’ and ‘hard of hearing’ to ensure a broad scope of the search and ensure that no potential studies were missed.

### 3.2 Eligibility Criteria

We ran preliminary searches to develop the eligibility criteria. Specific inclusion/exclusion criteria are defined by population, interventions, comparators and outcomes (PICO).

## 4 Inclusion Criteria

### 4.1 Population

We included studies of DMLs aged up to 18 years. We also included studies of carers or parents of DMLs aged up to 18 years. We included studies providing that at least 75% of participants were under 18, or provided separate outcome data. We excluded studies entirely about adults and those about clinician perspectives. We included studies of children and adolescents with permanent deafness, and excluded studies about bimodal bilinguals (i.e., use of a signed and spoken language).

### 4.2 Interventions

We included studies addressing factors which may affect the development of spoken expressive language, spoken receptive language, speech intelligibility, speech production, speech perception and lexical tone for DMLs.

### 4.3 Comparator

We included studies with no comparator, care as usual, alternative approaches to supporting the development of spoken expressive language, spoken receptive language, speech intelligibility, speech production, speech perception and lexical tone. Studies with any comparator or no comparator were included. Anticipated comparators included no intervention or care as usual. In the first instance, decisions did not use outcomes to guide inclusion/exclusion. Included papers reported interventions provided by practitioners, which facilitated speech and language development for DMLs. Studies reporting the effectiveness of any measures or interventions were also reviewed.

### 4.4 Outcomes

We included studies addressing outcomes including spoken expressive language, spoken receptive language, speech intelligibility, speech production, speech perception, lexical tone and studies including multilingualism. Where otherwise eligible studies did not report a relevant outcome, the researchers aimed to contact the authors to determine whether assessment of a relevant outcome occurred.

The primary outcome for the review is identification of factors affecting the listening, speech and language of DMLs. Secondary outcomes are (a) Changes to the listening, speech and language of DMLs; (b) Any identified barriers to and facilitators of listening, speech and language outcomes of DMLs.

### 4.5 Study Designs

We included quantitative studies with the following designs: randomized controlled trials and non‐randomized controlled studies, studies with pre and post intervention data, cohort studies, cross‐sectional studies and process evaluations. Also included were doctoral theses, mixed method studies and qualitative studies. We did not include case studies because it is not usual to include them in systematic reviews and they are limited by their retrospective, nonblinded and nonrandomized trial design, constituting a source of bias that could affect the study outcome. We did not include reports, opinion pieces, editorials, systematic and scoping reviews, but instead used reviews to identify any primary studies which were then added to the PRISMA.

## 5 Inclusion Screening

**FIGURE 1:**
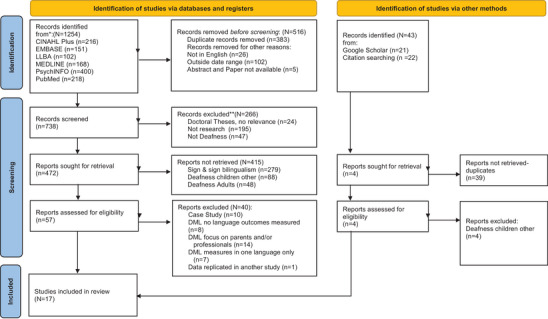
PRISMA 2020 flow diagram for new systematic reviews which included searches of databases, registers and other sources. *Consider, if feasible to do so, reporting the number of records identified from each database or register searched (rather than the total number across all databases/registers). **If automation tools were used, indicate how many records were excluded by a human and how many were excluded by automation tools. *Source*: Page MJ, et al. BMJ 2021;372:n71. https://doi.org/10.1136/bmj.n71. This work is licensed under CC BY 4.0. To view a copy of this license, visit https://creativecommons.org/licenses/by/4.0/.

To check for consistency, two reviewers independently screened an initial 100 references. Discussion achieved consensus and where this could not be reached, the third reviewer resolved any disagreements. Two researchers then independently assessed the evidence for inclusion using the eligibility criteria at both title and abstract and full text screening. Discussion achieved consensus where this could not be reached, the third reviewer resolved any disagreements. Reasons for exclusion are documented in Figure 1, the PRISMA flowchart (Page et al. 2021).

## 6 Tabulation of Data

We developed a bespoke data extraction form for data and piloted it using two researchers on a small set of studies representing the range of included study designs. Subsequently, one researcher extracted the data and another checked it. Tabulation of characteristics of the evidence included (a) Author and date, (b) Country, (c) Research design, (d) Sample size, (e) Percentage DMLs, (f) Age Range, (g) Home Language, (h) Languages assessed, (i) Measurement tools, (k) Outcomes, (l) Noted barriers and facilitators.

For quantitative outcomes, researchers aimed to extract data to permit the calculation of relative risks with 95% confidence intervals or mean or standard mean differences with 95% confidence intervals where appropriate. However, data did not provide a sufficient level of detail to support further analyses.

## 7 Assessment of Methodological Quality

The methodological quality of each research study included in the review was assessed. Assessing methodological quality is important because it helps to highlight issues in the objectives, design, data collection, analysis and conduct of research, with the aim of improving research methodology for the area and ultimately reducing studies which merely replicate existing research and fail to add or move the evidence base forwards.

Evaluation of studies included in the final data synthesis for methodological quality occurred. The studies were methodologically diverse and therefore two researchers (EK, JO) used the Quality Assessment with Diverse Studies (QuADS) tool (Harrison et al. 2021). QuADS displays strong content, face validity and inter‐rater reliability (Harrison et al. 2021). The tool assesses various important aspects of studies, such as the underlying theory, defined objectives, appropriateness and rigor of the design, data collection methods and analytical methods. It consists of 13 evaluative indicators (see Table 5), each rated on a four‐point Likert scale ranging from 0 (not at all) to 3 (complete), allowing researchers to determine the extent to which each criterion is met. To ensure consistency within the quality assessment, EK and JO carried out an initial pilot on 10% of the sample. They independently evaluated the quality of the studies and resolved any discrepancies through discussion. For the remainder of the papers, the third member of the review team (PC) resolved any conflicts of agreement (see Table 5 for further description and agreed scores).

## 8 Analysis and Synthesis

It was not possible to calculate effect estimates (RR, MD or SMD with 95% CI) for quantitative data because of limitations in the evidence. Therefore, we report on available measures of effect.

The quantity, quality, conceptual richness and contextual thickness of data in the evidence dictated using a narrative synthesis (Flemming et al., 2018; 2019). This is because data were ‘thin’ and indicated an elevated level of heterogeneity, limiting the choice of synthesis method to a narrative synthesis, supported by structured tables to tell the story of the findings from the included studies. The synthesis employs guidance for synthesis without meta‐analysis (SWiM) because of heterogeneity of the studies (Campbell et al. 2020; Popay et al. 2006).

## 9 Results

The PRISMA flowchart (Figure 1) illustrates the search results. Out of 1254 records, we documented reasons for exclusion in the PRISMA flowchart, leaving 17 studies for inclusion in the review.

### 9.1 Characteristics of Studies

We constructed a table for characteristics of the database evidence of peer‐reviewed papers comprising 17 papers (Table 1).

**TABLE 1:** Characteristics of included studies (N = 17).

Authors (year)	Country	Study design	Sample size	% DML	Age range	Home language	Languages assessed	Measurement tools	Outcomes	Barriers and facilitators
Bunta & Castilla‐Earls (2022)	USA	Longitudinal‐cohort	22	50	4‐7 years	Spanish	English, Spanish	Pre‐school Language Scale v.5 (PLS‐5) English / Spanish Word Intelligibility Picture Identification (WIPI) Test	The Bilingual TH group did better on PLS‐5 Spanish / English and WIPI scores at Time 1 and Time 2. Spanish maintenance was higher for bilingual TH group	Barrier to Spanish maintenance for DML group may be receipt of 'intensive therapy' in English and only 'Spanish support' as part of their intervention programme in USA
Bunta and Douglas (2013)	USA	Longitudinal‐cohort	40	50	Mono HL group Mean = 47.3 months s.d = 13.6 DML group Mean = 51.9 months s.d = 14.6	English, Spanish	English, Spanish	Monolingual HL group = Pre‐school language scale v. 4 (PLS‐4) English DML group = PLS‐4 English /Spanish	No difference in PLS‐4 English scores between monolingual HL and DML groups; no difference in English and Spanish PLS‐4 scores for DML group / strong correlation between English and Spanish PLS‐4 Total Language scores.	DML provided with dual language support achieved English PLS‐4 scores similar to monolingual HL peers. Target languages can both be acquired at similar levels. Encouraging use of Spanish at home was facilitator, discouraging mainstream language usage by non‐native parents
Bunta et al. (2016)	USA	Longitudinal‐cohort	20	100	3‐5 years	Spanish	English and Spanish	PLS‐4 English only reported	Dual language support group out performed English only support group on PLS‐4 Total Language and Expressive communication measure in English. No significant difference in Auditory Comprehension scores. Differences in Language Age matched this pattern	''Higher quality and quantity language input at home yields better speech and language outcomes in both languages'' p469. Supporting both the home language and the language of the majority culture potentially yields superior results compared to providing support only in the language of the majority culture.
de Diego‐Lázaro et al. (2021)	USA	Longitudinal‐cohort	73	18	8‐12 years	English, Spanish	English and Spanish	Adapted Receptive One‐Word Picture Vocabulary Test–4: Spanish‐Bilingual Edition. Word learning task, retention task.	Bilingual children did not differ significantly from monoling peers on the word learning tasks in both HL and TH groups. No significant difference between groups	Bilingualism was not a word learning advantage for children on word learning tasks. Vocabulary outcomes (English and Spanish) were similar across groups. Bilingual TH and DML all performed better on English rather than Spanish vocabulary, English /Spanish Word Learning ‐ no significant differences between groups
Forli et al. (2018)	Italy	Longitudinal‐cohort	28	50	2‐7years	Italian, Arabic, Chinese, Albanian, Romanian, Bengali, Spanish, Portuguese, Russian	Italian	Test Fono‐Lessicale (TFL), Test del Primo Linguaggio (TPL), Italian version of the Peabody Picture Vocabulary Test‐revised 3rd edition (PPVT‐R), One‐Word Picture Vocabulary Test, Test of Comprehension of Grammar for Children, (TCGB), Test of early verbal comprehension, AMBO kit, Ling Sound Test, Student Oral Language Observation Matrix (SOLOM).	No significant difference between DML and monolingual HL groups (using Italian measures); DML group scored better in lexical comprehension; DML generally scored lower language scores; SOLOM Italian ‐ DML had lower scores than Monolingual group (statistically significant); DML scored better on mainstream language than native language due to exposure to Italian at school, rehabilitation and home. DML with 'good bilingual exposure' and 'prevalent native language exposure' scored better SOLOM score on native language	Barrier to language acquisition ʻʻrelated to status of bilingual families in Italyʼʼ (p61) ʻʻlow socio‐economic and cultural levels…poorly integrated into their new home country…poor competence in Italian…difficulties following therapeutic and rehabilitative path.ʼʼ A barrier is services that are not centred on individual patients/ families. Low exposure to Italian language at home.
Guiberson (2014)	Spain	Longitudinal‐cohort	51	49	7‐8 years	English, German, Italian, Dutch, Gallego, Catalan, Basque, Spanish	Spanish, English, German, Italian, Dutch, Gallego, Catalan, Basque	SOLOM	DML children appeared to benefit from cross‐language transfer.	DML children demonstrated significantly stronger SOLOM L1 skills than their monolingual peers
Li et al. (2017)	USA	Longitudinal‐ cohort	54	22	4‐8years	Spanish, English	Spanish, English	Single‐word picture elicitation task was administered to the participants in Spanish or English	Being bilingual doesn't impact production qualities of CI users' fricative and affricative productions (within languages) Systematic support for CI users in Spanish and English lead to better Spanish production than bilingual TH peers	Having a CI was a barrier to differentiating some acoustic qualities. CI barrier to clear placement contrasts for both monolingual TH and DML.
Lund et al. (2015)	USA	Cohort (Pilot)	37	24	3‐6years	Spanish, English	Spanish and English	Test of Word Reading Efficiency (TOWRE), Rhyme Awareness subtest of the Phonological Awareness and Literacy Screening for Preschool (PALS‐PreK), Initial Sound Awareness subtest of the Phonological Awareness and Literacy Screening for Kindergarten (PALS‐K), Expressive and Receptive One Word Picture Vocabulary Tests (EOWPVT, ROWPVT, respectively) English and Spanish–English bilingual versions	Children with HL overall may develop phonological awareness differently from children without HL. Interventions should target skill development as it relates to a particular child's lexicon. Different pathways of hearing development may lead to varied literacy outcomes. The lack of correlation between vocabulary measures and phonological awareness in children with HL may reflect a measurement problem.	Bilingualism may provide a phonological awareness advantage for DMLs. The lexical restructuring model is valid for children without HL, but does not represent the way phonological awareness is acquired in children with HL.
McConkey Robbins et al. (2004)	USA	Analytical Cross‐sectional	12	100	20 months‐12 years	Hebrew, Yiddish, French, Spanish, Arabic, German, Armenian	English and child's Second Language	Reynell Developmental Language Scales (RDLS), Oral and Written Language Scales (OWLS), (translated to Yiddish for two children as 1^st^ language), SOLOM	Learning a second language did not impair L1 skills of CI users. Not significant.	Length of CI experience and amount of L2 exposure increased SOLOM scores. L2 proficiency is also facilitated by 1) parents speaking a second language at home 2) opportunities to use language two outside of home 3) wearing CI for extended time 4) adults using L2 are highly fluent in that language.
Sosa and Bunta (2019)	USA	Analytical Cross‐sectional	40	25	4‐7years	Spanish, English	Spanish, English	Age appropriate picture elicitation task, PLS‐5, modified version of the Inconsistency Assessment.	The use of two spoken languages did not appear to impact overall accuracy in English. Children with CIs had significantly higher rates of whole word variability than the children without CIs. Clinicians working with children who use CIs should consider incorporating treatment activities that target consistent production of individual words which may increase the robustness of phonological representations	Children with earlier CI had less whole‐word production variability. More exposure to language gives the child more opportunity to extract important information from the speech signal i.e. variability in exposure of each language leads to whole‐word production variability difference across languages
Teschendorf et al. (2011)	Germany	Analytical Cross‐sectional	93	56	3‐6years	Arabic, Albanian, Berber, Italian, Kurdish, Polish, Portuguese Serbo‐Croatian	Objective measures for German and SOLOM for home language	Mainzer test for speech comprehension in childhood, Göttinger Test for Speech Comprehension in Childhood Level I, Schmid‐Giovanini score for Listening stages, Pollack score for Talking stages, SOLOM	Children in the monolingual group demonstrated better scores than children in the bilingual group. Children with greatest exposure to second language at home did worst in German language tests	Barriers included immigration status, lower socioeconomic and education status, integration of family. Parent(s) not speaking German fluently. Twenty percent rule: exposure < 20% to language = child understands the language, but not want to speak.
Thomas et al. (2008)	USA	Longitudinal ‐cohort	24	50	NR	Arabic, Cantonese, English, French, Gujarati, Marathi, Spanish	English, Arabic, Spanish, French, Marathi, Gujarati, Cantonese	Infant Toddler Meaningful Integration scale (IT‐MAIS), PPVT, MacArthur Bates Communicative Development Inventory: Words and Gestures, OWLS, SOLOM	No significant difference on any measures at any interval between monolingual and bilingual groups. Variability in English and home language proficiency may be due to varied age of children, communication mode, use of English by parents	Facilitators included language rich environment in home language, parent empowerment to work regularly and frequently with child at home
van der Straten Waillet et al. (2025)	Belgium	Quasi‐experimental	69	22	4‐11 years Monoling TH group M=59.2 (s.d.=9.8) Monoling CI group M=88.2 (s.d.=22.0) Biling TH group M=64.7 (s.d.=4.8) DML group M=91.3 (s.d.=19.3)	DMLs = Portuguese, Turkish, Albanian, Aramaic, Czech, English, Mandarin, Persian, Russian, Spanish, Syrian Arabic Bilingual TH = English, Romanian, Turkish, German, Italian, Albanian, Egyptian Arabic, Greek, Lithuanian, Moroccan Arabic, Polish, Portuguese, Pulaar, Somali, Spanish	French and home language	Exalang 3‐6 (+4 additional items) for lower language level children, Evaleo 6‐15 (+7 additional items) for higher language level children Intelligibility in Context Scale	PCC: Bilingual and monolingual CI and TH obtained similar PCC (weak evidence) DML lower than bilingual TH (weak evidence) PVC: DML and monolingual CI similar PVC (weak evidence); DML and bilingual TH similar PVC (weak evidence); Speech Intelligibility: FRENCH ‐ DML similar to bilingual TH (weak evidence); DML similar to monolingual CI (weak evidence); HOME LANGUAGE ‐ DML lower than bilingual TH (moderate evidence)DML 73.3% highly intelligible in French / monolingual CI‐ 69.2% highly intelligible in French; DML‐ 46.2% highly intelligible in home language	Presences of CIs negatively impacted on intelligibility in home language (but not the community language) and consonant accuracy Lower linguistic dominance in home language reduced DML children's ability to achieve intelligible speech in home language
Vukkadala et al. (2018)	USA	Analytical Cross‐sectional	59	56	9.7 ± 3.0 ‐ 10.5 ± 3.2	Children from Hispanic and Asian cultures but no language mentioned apart from English	English	OWLS‐II Listening Comprehension (LC) and Oral Expression (OE) scales, SOLOM	DMLs are at increased risk for poor oral expressive and receptive language development.	Female sex, higher income, birth in USA ‐ significantly associated with better OWLS scores(English) Number of years exposed to English ‐ moderately correlated with better English OWLS scores
Waltzman et al. (2003)	USA	Analytical Cross‐sectional	18	100	NR	Armenian, English, French, Hebrew, Russian, Spanish, Yiddish	English and home language	Glendonald Auditory Screening Procedure (GASP), Phonetically Balanced Kindergarten test, Consonant‐Vowel‐Consonant test, Multisyllabic Lexical Neighbourhood Test and the Lexical Neighbourhood Test. Sentence Recognition: Common Phrases test and Bamford‐Kowal‐Bench test, Hearing‐in‐Noise test Language: RDLS (English) or OWLS (English)‐ Listening Comprehension Scale and Oral Expression Scale, SOLOM	Pre and post‐operative scores for speech perception statistically significant First Language results (English): Majority of children showed age‐appropriate receptive and/or expressive language abilities in their primary language (English). Second Language: no relationship between SOLOM rating and speech perception or language score in primary language	Living environment and parental influence appeared to be factors in second language learning; in addition to second language use at home, attendance at bilingual school, intervention in second language as well as first language. Barrier to second language learning was lack of exposure or late exposure to second language
Yim (2012)	USA	Longitudinal‐cohort	12	100	4‐8 years	English, Spanish	English, Spanish	PLS‐4, English and Spanish versions, PPVT‐IV, Test de Vocabulario en Imagenes Peabody (TVIP)‐Spanish, EOWPVT‐English and Spanish, Goldman‐Fristoe Test of Articulation‐2 (GFTA‐2) for articulation skills in English	English receptive and expressive scores and overall English semantic and syntactic scores of DMLs increased with age.	Facilitators: age, duration of implantation, amount of home language spoken and type of communication mode similar for bilingual TH and DML; Oral communication mode with increase amount of Spanish spoken at home Barrier: Total communication mode was a barrier for children's spoken Spanish language skills
Yorgancilar & Sizer (2022)	Turkey	Analytical Cross‐sectional	50	80.35	3‐6years	Arabic, Turkish, Kurdish	Unclear which language pre‐implant assessment completed in. Post completed in Turkish and Kurdish and just Turkish. Arabic /Turkish not assessed because of refugee status and length of time in the country.	Denver II Developmental Screening Test = pre‐implant speech and development Categories of Auditory Performance (CAP) Speech Intelligibility rating (SIR) Turkish version Test of The Early Language Development (TELD‐3); IT‐MAIS; Meaningful Use of Speech Scale (MUSS)	No significant difference between the groups in terms of the CAP and SIR scores. The Arabic/Turkish group had significantly lower scores. Exposure to a second language does not affect development of language during education.	Barrier: Refugee and asylum status of families; educational level of parents.

Abbreviations: CI = cochlear implant; DML = deaf and multilingual learner; HL = hearing loss; NR = not reported; L1 = language one; L2 = language two; PCC = percent consonants correct; PVC = percent vowels correct; TH = typical hearing

Research designs for the 17 peer‐reviewed papers comprised nine longitudinal studies (52.9%), one pilot study (5.8%) and six analytical cross‐sectional studies (35.3%) and one quasi‐experimental design (5.8%). Countries in the studies were: USA, 12 studies (70.5%), Italy, one study (5.88%), Spain, one study (5.88%), Germany, one study (5.88%), Turkey, one study (5.88%), Belgium, one study (5.88%). The level of diversity in the study designs meant sample sizes ranged from 12 to 93 participants with a median of 40.

Percentage of DMLs in the studies ranged from 100%– 4 studies (Bunta et al. 2016; McConkey Robbins et al. 2004; Waltzman et al. 2003; Yim, 2012), 80.4%‐1 study (Yorgancilar & Sizer, 2022), 56%‐2 studies (Teschendorf et al. 2011; Vukkadala et al. 2018), 50%‐4 studies (Bunta and Castilla‐Earls 2022; Bunta and Douglas 2013; Forli et al. 2018; Thomas et al. 2008), 49%‐1 study (Guiberson 2014), 25%‐1 study (Sosa and Bunta 2019), 24%‐1 study (Lund et al. 2015), 22%‐2 Studies (Li et al. 2017; van der Straten Waillet, 2025), 18%‐1 study (de Diego‐Lázaro et al., 2021).

Multilingualism included Spanish and English–8 studies (Bunta and Castilla‐Earls 2022; Bunta and Douglas 2013; Bunta et al. 2016; de Diego‐Lázaro et al., 2021; Li et al. 2017; Lund et al. 2015; Sosa and Bunta 2019; Yim, 2012), Italian, Arabic, Chinese, Albanian, Romanian, Bengali, Spanish, Portuguese, Russian‐1 study (Forli et al. 2018), English, German, Italian, Dutch, Gallego, Catalan, Basque, Spanish–1 study (Guiberson 2014), Hebrew, Yiddish, French, Spanish, Arabic, German, Armenian, but only English and the child's second language assessed–1 study (McConkey Robbins et al. 2004), Arabic, Albanian, Berber, Italian, Kurdish, Polish, Portuguese Serbo–Croatian, but only German and home language assessed–1 study (Teschendorf et al. 2011), Arabic, Cantonese, English, French, Gujarati, Marathi, Spanish–1 study (Thomas et al. 2008), French and Portuguese, Turkish, Albanian, Aramaic, Czech, English, Mandarin, Persian, Russian, Spanish, Syrian Arabic, Romanian, German, Italian, Egyptian Arabic, Greek, Lithuanian, Moroccan Arabic, Polish, Pulaar, Somali–1 study (van der Straten Waillet, 2025), Children from Hispanic and Asian cultures but no language mentioned apart from English–1 study (Vukkadala et al. 2018), Armenian, English, French, Hebrew, Russian, Spanish, Yiddish–English and home language assessed–1 study (Waltzman et al. 2003), Arabic, Turkish, Kurdish; Turkish not assessed because of length of time in country and refugee status centres–1 study (Yorgancilar & Sizer, 2022).

Studies employed 42 different measurement tools making it difficult to compare across studies. The diversity in outcome measurement tools reflects the heterogeneity within the sample population of each study. For example, different tools were employed to measure outcomes in children of different ages and language abilities and objective outcome measurement tools were matched to the spoken language being tested. We have described the outcome measurement tools used across the included studies in Table 2.

**TABLE 2:** Outcome measurement tools.

	Measurement tool	Domain assessed	Language of administration	Objective/ observational	Validated and standardised	CIs and/or degree of probability (p)	Studies which utilized tool
1	Pre‐school language scale (PLS‐4) english (Zimmerman et al. 2002)	Expressive and receptive oral language	English	Objective	Yes	Raw score, language age, total language score, subscale scores	Bunta et al. (2016) Bunta and Douglas (2013) Yim (2012)
	Pre‐school language scale v.5 (PLS‐5) English (Zimmerman et al. 2012)	Expressive and receptive oral language	English	Objective	Yes	Standardised score	Bunta and Castilla‐Earls (2022)
2	Pre‐school language scale (PLS‐4) Spanish (Zimmerman et al. 2002)	Expressive and receptive oral language	Spanish (English)	Objective	Standardised	Total language score	Bunta and Douglas (2013) Yim (2012)
	Pre‐school language scale v.5 (PLS‐5) Spanish (Zimmerman et al. 2012)	Expressive and receptive oral language	Spanish (English)	Objective	Standardised	Item responses, Standardised score	Bunta and Castilla‐Earls (2022)
3	Reynell developmental language scales (RDLS) (Reynell & Gruber, 1990)	Oral language comprehension and expression	English	Objective	Standardised	Standardised score	McConkey Robbins et al. (2004) Waltzman et al. (2003)
4	Oral and written language scales (OWLS) (Carrow‐Woolfolk, 1995)	Oral and written language	English	Objective	Standardised	Standardised score	McConkey Robbins et al. (2004) Thomas et al. (2008) Waltzman et al. (2003)
	Oral and written language scales (OWLS‐II) (Carrow‐Woolfolk, 2011)	Oral and written language	English	Objective	Standardised	Standardised score	Vukkadala et al. (2018) Waltzman et al. (2003)
5	Test of the early language development (TELD‐3:T)—Turkish version (Tophas & Guven, 2001)	Receptive and expressive language	Turkish	Objective	Standardised	Mean, standard deviation	Yorgancilar & Sizer (2022)
6	Receptive one‐word picture vocabulary test 4th edition—Spanish‐bilingual edition (ROWPVT‐4: SBE) (Martin & Brownell, 2010)	Receptive vocabulary (total acquired vocabulary)	English and Spanish	Objective	Standardised	Mean, standard deviation	de Diego‐Lázaro et al. (2021) Lund et al. (2015)
7	Peabody picture vocabulary test (PPVT‐R) (revised 3rd edition) Italian version (Stella, Pizzoli & Tressoldi, 2000)	Receptive vocabulary	Italian	Objective	Standardised	Mean	Forli et al. (2018)
8	Peabody picture vocabulary test (PPVT) 3^rd^ edition (Dunn & Dunn, 1997)	Receptive vocabulary	English	Objective	Standardised	Standardised score	Thomas et al. (2008)
	Peabody picture vocabulary test 4^th^ edition (Dunn & Dunn, 2007)	Receptive vocabulary	English	Objective	Standardised	Standardised score	Yim (2012)
9	Test de Vocabulario en Imagenes Peabody (TVIP) (Dunn et al., 1986)	Receptive vocabulary	Spanish	Objective	Standardised	Standardised score	Yim (2012)
10	Expressive one‐word picture vocabulary test 4th edition—Spanish‐bilingual edition (ROWPVT‐4: SBE) (Martin, 2010)	Expressive vocabulary (total acquired vocabulary)	English and Spanish	Objective	Standardised	Mean, standard deviation	Lund et al. (2015) Yim (2012)
11	One‐word picture vocabulary test (Brizzolara, 1989)	Lexical production	Italian	Objective	Standardised	Mean	Forli et al. (2018)
12	Test Fono‐Lessicale (TFL) (Vicari et al., 2007)	Lexical comprehension and production	Italian	Objective	Standardised	Mean	Forli et al. (2018)
13	Test del Primo Linguaggio (TPL) (Axia, 1995)	Lexical comprehension and production	Italian	Objective	Standardised	Mean	Forli et al. (2018)
14	Test of early verbal comprehension (Chilosi et al., 2003)	Language comprehension	Italian	Objective	Standardised	Mean	Forli et al. (2018)
15	Test of comprehension of grammar for children (TCGB) (Chilosi & Cipriani, 1995)	Grammatical comprehension	Italian	Objective	Standardised	Mean	Forli et al. (2018)
16	MacArthur Bates communicative development inventory: words and gestures (Fenson et al., 2006)	Word understanding and production	English and home language	Observational	Standardised	Mean	Thomas et al. (2008)
17	Word intelligibility picture identification (WIPI) test (Cienkowski et al. 2009)	Speech recognition	English	Objective	Standardised	Standardised score	Bunta and Castilla‐Earls (2022)
18	Protocollo Comune di Valutazione dei Risultati in Audiologia Riabilitativa (PCVRAR) (AA.VV., 1997)	Speech perception	Italian	Objective	Non‐standardised	% score	Forli et al. (2018)
19	Mainzer test for speech comprehension in childhood (Biesalski et al., 1974 in German; Chilla et al., 1976 author translation)	Word recognition	German	Objective	Standardised	% correct	Teschendorf et al. (2011)
20	Göttinger test for speech comprehension in childhood level I (Kollmeier & Wesselkamp, 1997)	Speech understanding	German	Objective	Non‐standardised	% correct	Teschendorf et al. (2011)
21	Glendonald auditory screening procedure (Erber, 1982)	Word recognition	English	Objective	Non‐standardised	% correct	Waltzman et al. (2003)
22	Phonetically balanced kindergarten test (Haskins, 1949)	Word recognition	English	Objective	Non‐standardised	% correct	Waltzman et al. (2003)
23	Consonant‐vowel‐consonant test (Peterson, 1962)	Word and phoneme recognition	English	Objective	Non‐standardised	% correct	Waltzman et al. (2003)
24	Multisyllabic Lexical neighbourhood test Lexical neighbourhood test (Kirk et al., 1995)	Monosyllable and multisyllabic word recognition	English	Objective	Non‐standardised	Total items correct	Waltzman et al. (2003)
25	Common phrases test (Robbins et al., 1988)	Sentence recognition	English	Objective	Non‐standardised	% correct	Waltzman et al. (2003)
26	Bamford‐Kowal‐Bench test (Bench et al., 1979)	Sentence recognition	English	Objective	Non‐standardised	% correct	Waltzman et al. (2003)
27	Categories of auditory performance (CAP) (Archbold et al., 1998)	Functional listening	Not specified	Observational	Non‐standardised	Rating score	Yorgancilar & Sizer (2022)
28	Schmid‐Giovannini score for ‘listening stages’ (Schmid‐Giovannini, 1997)	Stages of auditory perception	German	Information not available	Information not available	Information not available	Teschendorf et al. (2011)
29	Infant toddler meaningful integration scale (IT‐MAIS) (Zimmerman‐Phillips et al., 1998)	Auditory development and integration	English / home language	Observational	Non‐standardised	Rating score	Thomas et al. (2008) Yorgancilar & Sizer (2022)
30	Pollack score for talking stages (Pollack, 1985)	Expressive speech	German	Information not available	Information not available	Information not available	Teschendorf et al. (2011)
31	Meaningful use of speech scale (MUSS) (Robins & Osberger, 1990)	Use of voice, speech and communication strategies	Turkish, Kurdish	Observational	Non‐Standardised	Rating score	Yorgancilar & Sizer (2022)
32	Student oral language observation matrix (SOLOM) (Parker et al. 1985)	Communication competence	All languages	Observational	Non‐standardised	Rating score	Forli et al. (2018) Guiberson (2014) McConkey Robbins et al. (2004) Teschendorf et al. (2011) Thomas et al. (2008) Vukkadala et al. (2018) Waltzman et al. (2003)
33	Novel word learning task	Word learning	English Spanish Arabic (unfamiliar to all participants)	Objective	Non‐standardised	Mean, standard deviation	de Diego‐Lázaro et al. (2021)
34	Novel retention task	Word retention	English Spanish Arabic (unfamiliar to all participants)	Objective	Non‐Standardised	Mean, standard deviation	de Diego‐Lázaro et al. (2021)
35	Novel single‐word picture elicitation task	Speech production	Spanish, English	Objective	Non‐standardised	Acoustic analysis	Li et al. (2017)
36	Novel picture naming task	Speech production—accuracy and variability	Spanish, English	Objective	Non‐standardised	% Consonants correct	Sosa and Bunta (2019)
37	Exalang 3–6 – naming task (+4 additional items) (Helloin & Thibault, 2006)	Speech accuracy	French	Objective	Non‐standardised (Test used with additional items)	% Consonants Correct % Vowels Correct	van der Straten Waillet et al. (2025)
38	Evaleo 6–15 (+7 additional items) (Launay et al., 2018)	Speech accuracy	French	Objective	Non‐standardised. test used with additional items)	% Consonants correct % vowels correct	van der Straten Waillet et al. (2025)
39	Goldman‐Fristoe test of articulation‐2 (GFTA‐2) (Goldman & Fristoe, 2000)	Articulation	English	Objective	Standardised	Standardised score	Yim (2012)
40	Intelligibility in context scale (McLeod et al., 2012)	Speech intelligibility	French and home language	Observational	Non‐standardised	Rating score	van der Straten Waillet et al. (2025)
41	Speech intelligibility rating (SIR) (Cox & McDaniel, 1989)	Speech intelligibility	Not specified	Observational	Non‐standardised	Rating score	Yorgancilar & Sizer (2022)
42	Phonological awareness and literacy screening for preschool (PALS‐PreK), rhyme awareness subtest and initial sound awareness subtests (Invernizzi et al., 2004)	Phonological awareness	English	Objective	Non‐standardised	Mean, standard deviation	Lund et al. (2015)

Abbreviation: CI = confidence intervals.

The included studies varied in the inclusion of information about the communication interventions provided for the study participants with hearing loss (either monolingual children with hearing loss or DMLs). Typically children diagnosed with hearing loss in hearing centres are provided with access to interventions to support listening, speech and language development and the provision of the intervention affects communication outcomes. Two studies gave a detailed description of the intervention that participants accessed (2/17; 11.8%) and nine studies provided no intervention information (9/17; 52.9%). A summary of the included studies’ descriptions of communication interventions is provided in Table 3.

**TABLE 3:** Included studies’ description of communication intervention for participants with hearing loss.

**Study**	**Intervention type**	**Setting**	**Amount of intervention**	**Language of focus**
Bunta and Castilla‐Earls (2022)	Not described	Not specified	Not specified	Not specified
Bunta and Douglas (2013)	Auditory verbal therapy / auditory verbal education	All participants attended preschool: Centre for hearing and speech for one year	Group sessions in English – 3x a day, five days a week. Individual session one hour per week in English or Spanish	English for monolingual participants; English and Spanish (home language) for bilingual participants
Bunta et al. (2016)	Oral communication programme	Usual educational setting; preschool programme; day school for the deaf	All participants received the intervention for one year. Group or individual sessions for one hour per week	English only or English and Spanish (home language)
de Diego‐Lazaro et al. (2021)	Not described	Not specified	Not specified	Not specified
Forli et al. (2018)	Not described	Not specified	Not specified	Not specified
Guiberson (2014)	Not described	Not specified	Not specified	Not specified
Li et al. (2017)	Participants with CIs participated in oral‐aural intervention programmes	Not specified	Not specified	Not specified
Lund et al. (2015)	Oral communication	Not specified	Not specified	English
McConkey Robbins et al. (2004)	Oral communication	Not specified	‘intensive communication therapy’ (645)	Some participants had intervention in ‘second language’ (645)
Sosa and Bunta (2019)	All participants received Auditory Verbal Therapy	Centre for speech and hearing, private clinic, public school system	Not specified	Spanish and English
Teschendorf et al. (2011)	Not described	Not specified	Not specified	Not specified
Thomas et al. (2008)	Not described	Not specified	Not specified	Not specified
van der Straten Waillet et al. (2025)	Participants exposed to mix of communication approaches (i.e. oral, cued speech, sign)	Not specified	Not specified	Not specified
Vukkadala et al. (2018)	Not described	Not specified	Not specified	Not specified
Waltzman et al. (2003)	Not described	Not specified	Not specified	Not specified
Yim (2012)	All participants received speech‐language therapy	Private therapy through hospital	Not specified	Not specified
Yorgancilar & Sizer (2022)	Not described	Not specified	Not specified	Not specified

Abbreviation: CI = Cochlear implant

Numerous barriers and facilitators to listening, speech, and language development emerged (see Table 4), but support for more than one language and empowering parents appeared important.

**TABLE 4:** Barriers and facilitators to listening, speech and language development.

**Barriers**	**Facilitators**
Only one language used as part of intervention programme	DMLs provided with dual language support achieved scores similar to monolingual peers with hearing loss (HL).
Parents prioritising use of majority community language of country but lacking in proficiency	Supporting both the home language and the language of the majority culture potentially yields superior results compared to providing support only in the language of the majority culture
Services not centred on individual patient/ families.	Empowering parents to work frequently with children at home improves language development.
Having a cochlear implant (CI) is a barrier to producing differentiation of some acoustic qualities. CI barrier to clear placement contrasts for both monolingual HL and bilingual HL.	Earlier CI enables less whole‐word production variability. Earlier exposure to language gives the child more opportunity to extract important information from the speech signal
Lexical restructuring model is valid for children without HL but does not represent the ways children with HL acquire phonological awareness.	Increased speech accuracy supported reported speech intelligibility
Immigration status, lower socioeconomic and education status. Lack of family integration.	
Children understand the language but are resistant to speaking.	
Lack of exposure or late exposure to second language	

## 10 Quality of Studies

All studies included in this review employed quantitative methods, no qualitative studies emerged. Studies varied in methodological quality. No studies considered stakeholders in the design or conduct of the research.

Studies used a variety of comparison groups: DML and hearing multilingual children (2/17 12%); DML and deaf monolingual children (6/17; 35%); monolingual and multilingual hearing children and deaf monolingual children and DMLs (5/17; 29%); and groups of DMLs (4/17; 24%). This again made comparison difficult.

Nearly half of the studies included, provided a clear explanation of the theories or concepts which informed the research (8/17; 47.1%) and two studies provided a brief outline of their underpinning theories (2/17; 11.7%) (See Table 5). Thirteen studies included a clear statement outlining the research aims (13/17; 76.5%). All studies described the focus population, however, not all gave a detailed description of the research setting (9/17; 52.9%).

**TABLE 5:** Quality Assessment of the included studies based on QuADS (Harrison et al. 2021).

**Author / Year**	**Item 1**	**Item 2**	**Item 3**	**Item 4**	**Item 5**	**Item 6**	**Item 7**	**Item 8**	**Item 9**	**Item 10**	**Item 11**	**Item 12**	**Item 13**	**Score/39**	**%**
Bunta and Castilla‐Earls (2022)	3	3	2	2	2	3	3	3	2	3	3	0	2	31	79.48
Bunta and Douglas (2013)	3	3	3	2	3	2	3	2	1	1	3	0	2	28	71.79
Bunta et al. (2016)	2	3	3	2	2	2	3	2	1	3	2	0	2	27	69.23
de Diego‐Lázaro et al. (2021)	3	3	2	3	2	2	3	3	1	2	2	0	1	27	69.23
Forli et al. (2018)	2	2	3	3	2	2	3	2	1	0	3	0	1	24	61.53
Guiberson (2014)	3	3	1	2	2	3	2	2	2	2	2	0	3	27	69.23
Li et al. (2017)	3	3	3	3	1	3	3	3	2	1	3	0	2	30	76.92
Lund et al. (2015)	3	3	3	3	3	1	1	3	2	2	3	0	2	29	74.35
McConkey Robbins et al. (2004)	1	2	2	3	1	1	2	3	2	3	1	0	1	22	56.41
Sosa and Bunta (2019)	3	3	3	3	1	2	3	2	2	2	3	0	2	29	74.35
Teschendorf et al. (2011)	2	3	3	3	1	3	3	2	2	3	3	0	1	29	74.35
Thomas et al. (2008)	1	2	2	1	1	1	2	1	1	0	1	0	1	14	35.89
van der Straten Waillet et al. (2025)	3	3	3	2	2	3	2	3	1	0	2	0	2	26	66.67
Vukkadala et al. (2018)	2	3	3	3	2	2	3	2	1	2	2	0	2	27	69.23
Waltzman et al. (2003)	2	3	3	3	1	3	2	3	1	2	2	0	0	25	64.10
Yim (2012)	2	3	2	3	2	3	2	2	1	2	2	0	3	27	69.23
Yorgancilar & Sizer (2022)	2	2	2	1	1	1	2	1	1	1	0	0	1	15	38.46

Coding reference included in Supplementary Table 1.

Ten studies were rated as having used the most appropriate study design to answer the research questions posed (10/17; 58.8%). Five studies may have employed a more suitable study design (5/17; 29.4%) and two may have more fully answered their research questions with alternative designs (2/17; 11.8%). Sampling within fifteen studies lacked a detailed description of their sampling procedure and sample size considerations to support statistical power (15/17; 88.2%) while only two of the studies fulfilled these criteria (2/17; 11.8%).

Studies employed a wide range of data collection tools. Seven studies provided a clear and detailed rationale for the choice of collection tools (7/17; 41.2%) and four provided limited rationale (4/17; 23.5%). Nine studies employed tools supporting detailed data collection (9/17; 52.9%). Seven studies described data collection in detail (7/17; 41.2%). Over half of the studies provided limited detail of recruitment plans and outcomes (10/17; 58.8%).

There was a high level of variation in the provision of justification for the analytical method used within each study. Four studies provided detailed justification (4/17; 23.5%) and six studies provided a limited or no justification (6/17; 35.9%). Seven studies used the most appropriate analytical method for the stated research aims (7/17; 41.2%).

Finally, only two studies provided a critical analysis of the study strengths and limitations, with detailed explanation (2/17; 11.8%) and seven studies provided limited or no discussion of study strengths and limitations (7/17; 41.2%).

Risk of bias (RoB) was difficult to assess for numerous reasons. Considering the heterogenous evidence base, we could not conduct a sensitivity analysis and meta‐analysis, and there was no established reliable and valid fit‐for‐purpose tool for assessing RoB. Furthermore, the evidence did not offer a clear rationale to support a conclusion about bias. However, the studies all lacked a detailed analysis plan, making it impossible to determine if outcomes were selectively reported based on favourable results. We could therefore imply that all studies exhibited selective outcome reporting bias.

## 11 Discussion

This study carried out a systematic review of the factors affecting listening, speech and language outcomes for DMLs in each language heard and spoken by the child. Methodological quality of the evidence retrieved was variable and the ability to compare studies was not possible. No studies used interdisciplinary research which could potentially add insights in this area. In this review, the evidence retrieved determined facilitating factors and those acting as barriers to communication competence in more than one spoken language. The studies included languages spoken, including the majority community language alongside a different, home language. This study adds to the existing evidence base by drawing together from a range of existing studies, the factors that enhance or inhibit multilingual spoken language development in DMLs.

Factors found to enhance home language development, alongside developing the majority community language, included the provision of interventions in the home language and the community language (Bunta et al. 2016; Li et al. 2017). Other factors of importance included a language rich environment and amount of language exposure for DMLs (de Diego‐Lazaro, 2021; McConkey Robbins et al. 2004; Sosa and Bunta 2019; Thomas et al. 2008). Furthermore, the provision of interventions in the home language specifically enhanced home language outcomes (Bunta and Douglas 2013; Waltzman et al. 2003). This was also the case for the use of the home language in the educational setting (Waltzman et al. 2003), the level of exposure to the home language, and encouraging the use of the home language in the home over the majority community language (Bunta and Douglas 2013; Waltzman et al. 2003).

The factors that hindered development of the home language included interventions focusing only on the majority community language. Bunta and Castilla‐Earls (2022) commented that Spanish retention in Spanish and English speaking DMLs reduced when delivering interventions solely in English. A further barrier to DMLs’ outcomes in any of their languages was parental use of a majority community language when they lacked fluency as speakers (Teschendorf et al. 2011). This limited the benefits of ‘higher quality and quantity language input at home’ (Bunta et al. 2016, 469) because parents were unable to provide their child with a model of well‐structured and fluent language. Lastly, a lack of communication partners in the home language outside the home also inhibited language competence in the home language (McConkey Robbins et al. 2004). This is one area of potential for educators of DMLs.

Reporting of the languages spoken by DMLs, and their families occurred in all but one study. However, studies appeared less consistent or detailed about the exposure level for each child in each of the languages reported. Exposure to a language rich environment is a crucial pre‐requisite to attainment in fluency, recall and understanding (American‐Speech‐Language‐Hearing‐Association 2024). Researchers described language exposure in a number of ways in the evidence retrieved. For example, some studies included a percentage of language exposure; a rating based on parental reported or set criteria to meet in order to be included as a DML (i.e., age and length of exposure to two languages). Other studies omitted specific measures; instead, researchers provided broad descriptions for example, ‘exposed to a second language with varying amounts of intensity’ (McConkey Robbins et al. 2004, 645). The variation in reporting, as well as the lack of objectivity and variability in the degree of exposure to the language each child receives, made it difficult to draw comparisons and interpret the outcomes for DMLs. Employment of a standard language exposure measure may facilitate comparison of subjects within studies as well as across each study. For example, use of a tool such as Q‐BEx (Quantifying Bilingual Experience) which measures language exposure and use, could be employed by researchers and would allow for comparisons across different research studies (De Cat et al. 2022). Use of consistent and standardised descriptions of child characteristics could also facilitate comparison across studies. Of the studies included in this review the variability in reporting the age of diagnosis or age of implantation or fitting of amplification (6/17 reported age of diagnosis; 7/17 reported age of implantation or hearing aid fitting; 2/17 reported both; 1/17 reported length of device use only and 1/17 reported all three characteristics) limited meaningful comparison. Six studies gave detailed descriptions of the type and severity of deafness and the type of amplification used. Nine studies gave this detail in the context of all children being cochlear implant users, leaving readers to merely assume the severity of deafness using their existing knowledge of the audiological criteria for cochlear implantation.

The studies used 42 different tools to measure the listening, speech and language outcomes of DMLs. Although this variety is not unusual for the discipline, given the age range and communication competence of children included in the studies, it also limits comparison and generation of new insights, restricting development in the area. The languages and the availability of standardised and norm referenced tools for each language informed the selection of assessment tools used. In clinical practice, practitioners are required to provide care to families who speak a range of different languages, but standardised tools are not available in every language. This presents a challenge for practitioners. Seven studies used the Student Oral Language Observation Matrix (SOLOM) to assess DMLs’ competency in their home language. The SOLOM is a rating scale used to assess competency across five parameters: listening comprehension, vocabulary, fluency, grammar and pronunciation (Parker et al. 1985). While it is subjective in nature, it is a reliable and valid tool, with the potential to provide an opportunity for comparison across studies (Guiberson 2014).

An important factor to consider for all stakeholders in the development of listening, speech and language skills in DMLs, is language prestige and the perception of language status among researchers, practitioners, educators and the families of DMLs. Language ideologies (that is, the beliefs and attitudes about individual languages, language prestige and multilingualism) influences the way that the use of a particular language and multilingualism is encouraged (Ronderos et al. 2022). Migration and globalisation have increased the linguistic diversity in countries globally, with particular impact on countries with a dominant majority community language (e.g., English in the United Kingdom, United States and Australia; French in France and German in Germany). The evidence in this review suggests that within these communities, there is a perceived language hierarchy where the language of the mainstream community and of education is more valuable than minority migrant or indigenous languages. The perception of lesser value of minority or indigenous languages can lead to subtractive bilingualism. In the context of the DMLs observed in the studies of this review, as the child learns more of the majority community language their competence in the minority language would reduce, increasing the likelihood of inhibited family relationships or intergenerational conflict (Nguyen 2022). Providing guidance for educators in the importance of using minority or indigenous languages to assist learners would appear useful. Moreover, the provision of equitable and culturally sensitive research, which includes clinical attention to all languages of importance for families of DMLs, prevents researchers and practitioners presenting unconscious bias in the form of judgments about language value and its importance to DMLs and their families.

Parental and family engagement in intervention programs influenced listening, speech and language outcomes for DMLs (Thomas et al. 2008). Parental language skills in the majority community language influenced the level of engagement (Forli et al. 2018; McConkey Robbins et al. 2004; Teschendorf et al. 2011). However, level of engagement also related to socio‐economic or migration status and depended on level of education and parental linguistic skills (Forli et al. 2018; Teschendorf et al. 2011; Yorgancilar and Sizer, 2022).

This review found limited, but insightful evidence suggesting that parental empowerment facilitates outcomes for DMLs. This is confirmed by an isolated small‐scale study about DHH children, but not specifically for DMLs (Erbasi et al. 2018). There is therefore little evidence to guide educators and practitioners alike about improving outcomes for DMLs. Researchers facilitated empowerment by involving parents in child amplification use and involving parents in their child's listening, speech and language development. However, parental involvement is multifaceted and incorporates a broad range of behaviours and practices. This is because the work of parents takes place away from the context of the intervention to facilitate their child's speech and language development. For example, parents act as case managers for their child and may always have their language development at the forefront of their minds. They also act as advocates for their children and for the children of others through parent support groups (Moeller et al. 2024). The impact of the number of children in the household and extent of resources parents can draw upon, affects parental capacity to facilitate their child's learning, speech and language development. Parents have numerous roles, including their main role of parent, which they perform in differing contexts. However, acknowledgement of the expertise of parents and the numerous roles they perform appears important for practitioners to include in guiding interventions for DMLs and for including educators to extend learning and facilitate language development (Crowe and Guiberson 2021). Without support for parents, barriers to acquiring language skills for DMLs occur when intervention practices lack family centeredness, or a whole child approach (Forli et al. 2018). Practitioners can support family centred practice by including parents and their children in building interventions and communicating with parents in their preferred (and most proficient) language.

Successful family relationships depend upon communication between family members. For a DML whose deafness impacts upon their spoken language learning and who may not be supported to use the spoken language of the home, they may find it difficult to form deep and meaningful relationships with family members, influencing their wellbeing, mental health, sense of identity and shared culture. No studies discussed the socio‐emotional development and needs of DMLs, and this would appear to be an area in need of more exploration. This area of research would also benefit from the inclusion of qualitative research that seeks to explore the experiences of DMLs and their families, with particular focus on factors that enhance the development of spoken language multilingualism, family connectedness and wellbeing. The socio‐emotional outcomes such as well‐being, mental health and family connectedness could be explored through the use of questionnaires such as the family connectedness scale (Eisenberg and Resnick 2006), Stirling children's wellbeing scale (Liddle and Carter, 2015) and the Warwick‐Edinburgh mental well‐being scale (Clarke et al. 2011) and the insights gained through high quality qualitative research could contribute to culturally sensitive best practice guidelines. The provision of coordinated support involving parents, health practitioners, other families and educators has the potential to offer a more joined up approach towards facilitating the acquisition of more than one spoken language for DMLs.

Another important and unexplored area in the evidence is the use of culturally appropriate assessment and therapeutic materials, alongside including parents and their children in the development of interventions.

Finally, scrutiny of potential bias and assumptions based on an idealisation of ‘White, middle‐class trajectories’ (Holcomb et al., 2024, 5) has revealed insights to inform further research on the communication outcomes for DMLs. To the best of our knowledge, no studies had first authors who were deaf or hard of hearing, nor did any papers consult any deaf or hard of hearing individuals. Additionally, 41% of the included studies made comparisons between deaf and hearing children. This introduces bias related to the medicalisation of deafness and disregard of the linguistic strengths within the deaf community (Holcomb et al., 2024). By comparing DMLs to children with typical hearing as control groups, the implicit assumption that the spoken language of typically hearing children is the ideal rather than affirming the diverse communication skills within the deaf community (signed and/or spoken).

## 12 Limitations of the Study

This study used a systematic review for its design; the strength of this systematic review is that it follows the Cochrane guidance to ensure the methodology was robust and systematic. A systematic and comprehensive search of the evidence occurred, and the reporting of the search strategy followed the requirements of the PRISMA statement. Opportunities for pooling data for meta‐analysis were explored but limited because of heterogeneity in the evidence. Therefore, researchers used a narrative synthesis to present conclusions. The study did not assess between‐study heterogeneity quantitatively because the evidence precluded conducting a meta‐analysis. Inclusion of evidence meant researchers engaged in lengthy discussions to ensure they reached the most appropriate decision and fit for the study. Publication bias did not appear in this review because the majority of studies contained small sample sizes and failed to demonstrate statistically significant results. This study excluded research published in languages other than English. As a result, research may have been excluded from our search that could have contributed the final findings of the review. Repeated reviews on this topic should seek to acquire the resources to allow inclusion of research published in languages other than English.

All authors of this review are hearing, White and part of the linguistic majority within the United Kingdom and Republic of Ireland. We did not have consultation from deaf or multilingual individuals, which may have introduced unintended bias into the design of the review.

## 13 Conclusion

Future studies for DMLs should give equal weighting to the measurement of outcomes in each language regardless of community status. Researchers could address this challenge through collaboration across research centres nationally and internationally to seek the appropriate linguistic expertise. Furthermore, there is a need to include educational practitioners as people who are involved in language acquisition and development. For practitioners, this review provides valuable insight into how the listening, speech, language and communication skills of DMLs can be maximised by including parents to guide and facilitate the intervention. Furthermore, designing services that allow for multilingual assessment and intervention practices, valuing and embracing the cultural and linguistic identities of DMLs and their families moves speech and language therapy and associated practitioners towards a more equitable and inclusive form of service provision.

## Funding

E. Kilmartin held a part‐time Pre‐doctoral Fellowship funded by the National Institute for Health and Care Research Applied Research Collaboration Greater Manchester (NIHR ARC‐GM) (Grant award number NIHR200174). The views expressed are those of the authors and not necessarily those of the NHS, the NIHR, the Department of Health and Social Care, or its partner organisations.

## Conflicts of Interest

The authors disclose no conflicts of interest.

## Supporting information




**Supporting Information**: jlcd70191‐sup‐0001‐SuppMat1.docx

**Supporting Information**: jlcd70191‐sup‐0002‐SuppMat2.docx
