# Supplementary material for: Factors Affecting Communication Outcomes for Deaf and Multilingual Learners: A Systematic Review
Source: Int J Lang Commun Disord. 2026 Jan 28;61(2):e70191. doi: 10.1111/1460-6984.70191 (PMC12852977; doi:10.1111/1460-6984.70191)
Supplement: Supplementary file 1 — Supporting Information: jlcd70191‐sup‐0001‐SuppMat1.docx [file JLCD-61-0-s002.docx]

**Supplementary Document 2: Included Studies’ Outcome Measurement Tool References**

A.A. V.V. (1997). Protocollo Comune di Valutazione in Audiologia Riabilitativa, CRO Edition.

Archbold, S., Lutman, M. E., & Nikolopoulos, T. (1998). Categories of auditory performance: inter-user reliability. *British journal of audiology*, *32*(1), 7-12.

Axia, G. (1995). Test del primo linguaggio. *Firenze (Italy): Organizzazioni Speciali*.

Bench, J., Kowal, A., & Bamford, J. (1979). *The BKB (Bamford-Kowal-Bench) sentence lists for partially-hearing children.* Br J Audiol. 13(3),108-12. doi: 10.3109/03005367909078884. PMID: 486816.

Biesalski, P., Leitner, H., Leitner, E., & Gangel, D. (1974). Der Mainzer Kindersprachtest. Sprachaudiometrie im Vorschulalter [The Mainz audiometric speech test for children (author's transl)]. HNO. 22(5),160-1. German. PMID: 4842952.

Brizzolara, D. (1989). Test di vocabolario figurato. *Università di Pisa: IRCCS “Stella Maris*.

Carrow-Woolfolk, E. (1995). *Oral and Written Language Scales*. Circle Pines, MN: American Guidance Service, Inc.

Carrow-Woolfolk, E. (2011). *Oral and Written Language Scales*. Los Angeles, CA: Western Psychological Services.

Cienkowski, K. M., Ross, M., & Lerman, J. (2009). The Word Intelligibility by Picture Identification (WIPI) Test Revisited. *Journal of Educational Audiology*, *15*, 39-43. [4-article-09-libre.pdf](https://d1wqtxts1xzle7.cloudfront.net/85531252/4-article-09-libre.pdf?1651752082=&response-content-disposition=inline%3B+filename%3DThe_Word_Intelligibility_by_Picture_Iden.pdf&Expires=1757066407&Signature=If6eAzYtQyHEwlB8rv89xm9XEHkR~8I2INGq7al9nJjmar7ZL9nvEneUvZxfQAfKipvFZyz2qrJEu0pFmR5cIf86NY75OdZXN3LmUQovCx0PjNdUC~Kzvfon9DGE1B-Pyj8~JpwE99fraD8FYhqgoFjC9oxXRszW9ROk7omokA6bDqlFJpxQ6eUcN9XTi6BNfE3Kyu140IuBPyxZSiZ9Wjf7IF-QQSD19Jg91jV4GjuW6t2hDr9BFeEKRUN-u1jhqxguSOFEmLyEPThEG3Rx~zIQaUGZu~0~Undb67u8em2uoWKZU4APzszHmEO3OkwaqFXe8FFfVSifa~481-dqwQ__&Key-Pair-Id=APKAJLOHF5GGSLRBV4ZA)

Chilosi, A.M., & Cipriani, P. (1995) Test di Comprensione grammaticale per bambini. Edizioni del Cerro, Tirrenia.

Chilosi, A. M., Cipriani, P., Villani, S., & Pfanner, L. (2003). Capire giocando: uno strumento per la valutazione verbale precoce (TCVP). In *Technical Report, Italian National Research Council*.

Cox, R. M., & McDaniel, D. M. (1989). Development of the Speech Intelligibility Rating (SIR) test for hearing aid comparisons. *Journal of speech and hearing research*, *32*(2), 347–352. https://doi.org/10.1044/jshr.3202.347

Dunn, L. M. , Padilla, E., Lugo, D., & Dunn, L. M. (1986). Test de Vocabulario en Imágenes Peabody: Adaptación Hispanoamericana (TVIP). AGS.

Dunn, L. M., & Dunn, L. M. (1997). *Peabody Picture Vocabulary Test--Third Edition (PPVT-III)* [Database record]. APA PsycTests.

Dunn, L. M., & Dunn, D. M. (2007). *Peabody Picture Vocabulary Test--Fourth Edition (PPVT-4)* [Database record]. APA PsycTests

Erber, N. (1982). Glendonald auditory screening procedure. *Auditory training*, *47*(71).

Fenson, L., Marchman, V. A., Thal, D. J., Dale, P. S., Reznick, J. S., & Bates, E. (2006). *MacArthur-Bates Communicative Development Inventories, Second Edition (CDIs)* [Database record]. APA PsycTests.<https://doi.org/10.1037/t11538-000>

Goldman, R., & Fristoe, M. (2000). *Goldman-Fristoe Test of Articulation, Second Edition (GFTA-2)* [Database record]. APA PsycTests.<https://doi.org/10.1037/t15098-000>

Haskins, H. L. (1949). *A phonetically balanced test of speech discrimination for children* [Dissertation].

Helloin, M. C., & Thibault, M. P. (2006). Exalang 3-6. Ortho-Mothus.

Invernizzi, M., Meier, J., & Swank, L. (2004). *Phonological Awareness Literacy Screening for Preschoolers (PALS-PreK)* [Database record]. APA PsycTests.<https://doi.org/10.1037/t27727-000>

Kirk, K.I., Pisoni, D.B., & Osberger, M.J. (1995). Lexical effects on spoken word recognition by pediatric cochlear implant users. *Ear Hear* 16,470–481.

Kollmeier, B., & Wesselkamp, M. (1997). Development and evaluation of a German sentence test for objective and subjective speech intelligibility assessment. *The Journal of the Acoustical Society of America*, *102*(4), 2412-2421.

Launay, L., Maeder, C., Roustit, J., & Touzin, M. (2018). EVALEO 6-15. OrthoEditions.

Martin, N. A. (2010). *Expressive One-Word Picture Vocabulary Test 4th Edition - Spanish-Bilingual Edition*. Academic Therapy Publications.

Martin, N. A., & Brownell, R. (2010). *Receptive One-Word Picture Vocabulary Test 4th Edition - Spanish-Bilingual Edition*. Academic Therapy Publications.

McLeod, S., Harrison, L.J., & McCormack, J.  (2012). The intelligibility in context scale: validity and reliability of a subjective rating measure. *Journal of Speech, Language and Hearing Research,* 55(2),648-56. doi: 10.1044/1092-4388(2011/10-0130).

Parker, D., Dolson, D., & Gold, N. (1985). *Student Oral Language Survey (SOLOM)*. Sacramento: Bilingual Education Office of the California Department of Education. Retrieved from <http://www.cal.org/twi/EvalToolkit/appendix/solom.pdf>.

Peterson, G. E., & Lehiste, I. (1962). Revised CNC lists for auditory tests. *Journal of Speech and Hearing Disorders*, *27*(1), 62-70.

Pollack, D. (1985). *Educational Audiology for the Limited Hearing Infant and Pre-schooler*. Washington, DC: AG Bell.

Reynell, J., & Gruber, C. (1990). *Reynell Developmental Language Scales: US edition*. Los Angeles: Western Psychological Services.

Robins, A., Renshaw, J., & Osberger, M. (1988). *Common Phrases (test).*  Indianapolis, IN: Indiana University School of Medicine.

Robbins, A.M., & Osberger, M.J. (1990). *Meaningful Use of Speech Scale (MUSS).* Indianopolis: Indiana University School of Medicine.

Schmid-Giovannini, S. (1996). Hören und Sprechen. Anleitung zur auditivverbalen Erziehung hörgeschädigter Kinder. Stiftung Zentralstelle der Studentenschaft der Universität Zürich. Zürich.

Stella, G., Pizzoli, C., & Tressoldi, P. E. (2000). Peabody Picture Vucabulary Test PPVT-R Italian Standardization, Omega edition.

Tophas, S., & Guven, S. (2011). Test of The Early Language Development (TELD-3:T) – Turkish Version. Ankara: Detay Press in Turkish.

Vicari, S., Marotta, L., & Luci, A. (2007). *TFL Phono-lexical Test.: Assessment of lexical skills in preschool age*. Erickson Editions.

Zimmerman, I. L., Steiner, V. G., & Pond, R. E. (2002a). *Preschool Language Scale, Fourth Edition* (English Edition). San Antonio, TX: Harcourt Assessment.

Zimmerman, I. L., Steiner, V. G., & Pond, R. E. (2002b). *Preschool Language Scale, Fourth Edition* (Spanish Edition). San Antonio, TX: Harcourt Assessment.

Zimmerman, I. L., Steiner, V. G., & Pond, R. E. (2012). *Preschool Language Scale, Fifth Edition.* Pearson.

Zimmerman-Phillips, S., Robbins, A. M., & Osberger, M. J. (2000). Assessing cochlear implant benefit in very young children. *The Annals of Otology, Rhinology & Laryngology*, *109*(12), 42.
